# Sex Differences in Freezing Behavior and Extinction Learning Among Wistar Rats Exposed to Social Isolation and 15 cGy GCRsim Space Radiation Both Alone and in Combination

**DOI:** 10.3390/life16101638

**Published:** 2026-09-30

**Authors:** Zachary N. M. Luyo, Riley S. Heerbrandt, Alea F. Boden, Frederique E. E. Keumeni, Richard A. Britten, Laurie L. Wellman, Larry D. Sanford

**Affiliations:** 1 Sleep Research Laboratory, Department of Biomedical and Translational Sciences, Eastern Virginia Medical School, Macon and Joan Brock Virginia Health Sciences at Old Dominion University, Norfolk, VA 23507, USA; luyozn@odu.edu (Z.N.M.L.); riley.heerbrandt@gmail.com (R.S.H.); bodenaf@odu.edu (A.F.B.); keumenf@odu.edu (F.E.E.K.); wellmall@odu.edu (L.L.W.); 2 Center for Integrative Neuroscience and Inflammatory Diseases, Eastern Virginia Medical School, Macon and Joan Brock Virginia Health Sciences at Old Dominion University, Norfolk, VA 23507, USA; brittera@odu.edu; 3 Department of Radiation Oncology, Eastern Virginia Medical School, Macon and Joan Brock Virginia Health Sciences at Old Dominion University, Norfolk, VA 23507, USA

**Keywords:** social isolation, space radiation, extinction learning, stress-induced hyperthermia, sex differences

## Abstract

Long-term space missions traveling beyond the Earth’s magnetosphere will expose astronauts to prolonged periods of social isolation (SI) and exposure to space radiation (SR). Throughout these missions, astronauts will have to cope with these stressors and adapt to their environment to carry out mission objectives. It is known that SI and SR can impact mission performance and overall health. Additionally, behavioral stress responses can differ between sexes. However, the independent and interactive effects of SI and SR on fear behavior and extinction learning between sexes is unknown. This study compared freezing behavior and stress-induced hyperthermia (SIH) in female and male Wistar rats using a conditioned fear (CF) paradigm. Comparisons were made in SHAM-treated rats and in ground-based models of SI and SR alone and in combination (dual flight stressors (DFSs)). SHAM and SI females froze significantly more during shock training (ST), context re-exposure (CTX) and extinction learning (EXT) compared to their male counterparts, suggesting sex differences in fear learning and memory. There were minimal changes in freezing behavior between sexes in SR and DFS animals; however, SR and DFS females had significantly lower temperatures than their male counterparts. These findings suggest the presence of sex differences in stress responses, and that stress-based learning and memory can be differentially impacted by spaceflight hazards. Baseline differences suggest that males and female rats may have sex-specific characteristics attuned to their behavioral capacities or needs, whereas differences after exposure to spaceflight hazards suggest differences in stress vulnerability. In general, these data suggest that understanding within-sex alterations will be important for assessing the effects of spaceflight hazards on performance and developing mitigation strategies that may need to be tailored for males and females.

## 1. Introduction

Since the year 2000, there has been a continual human presence in space through the International Space Station. With the proposed Mars missions, astronauts will spend even greater time in space and will be exposed to several known spaceflight hazards, including longer periods of isolation and confinement along with potential social isolation (SI), larger dosages and different spectra of space radiation (SR), as well as possible interactions between hazards. SI and SR alone, and in combination (dual flight stressors (DFSs)), have a variety of effects on sensorimotor performance [1,2], brain structure [3] and inflammation [4,5,6] that could impact the ability of astronauts to perform their mission duties as well as manage potential emergencies that lie outside their normal duties.

Both SI and SR have been demonstrated to have differential effects in males and females. Most evidence suggests that women are lonelier than men [7,8] and that loneliness (perceived isolation) is associated is associated with significant health risks [9]. Additionally, research on SR has shown sex differences in microglial activation, synaptic modifications, and cognition following high-LET particle irradiation [10,11,12]. Interestingly, the effects of SR on behavioral assessments of conditioned fear and context discrimination appear to vary with radiation type (i.e., ^4^He, ^28^Si, ^56^Fe, GCRsim), dosage, and the sex of subjects [13,14,15,16]. Both SI and SR have been shown to exacerbate the body’s stress response, indicated by alterations in endocrine function [17,18,19]. Additionally, females are more vulnerable to stress- and fear-based psychiatric disorders (e.g., anxiety and posttraumatic disorder [20] and depression [21]) and are more susceptible to several neuroinflammatory diseases [21]. While the combination of SI and SR can produce interactive effects on the neuroimmune system and sensorimotor function [1,22,23,24], it is still unclear how SI and SR interact to influence several components of behavior and cognition. Thus, there is a need to understand how SI, SR and their interactions may differentially affect males and females, and how they may impact their ability to cope with unexpected challenges that may occur during spaceflight.

In this project, we utilized conditioned fear (CF), a common paradigm used in rodent models [3,25,26,27], to model how spaceflight hazards could impact responses to significant unexpected stress. CF is an important type of stress-related learning that can facilitate successful responses to future challenges but can be maladaptive and even result in pathological diseases like PTSD [28]. It occurs when a neutral stimulus or context becomes associated with a significant aversive emotional event (typically inescapable shock (IS) in standard experimental paradigms). Subsequently, exposure to these previously neutral stimuli and contexts can elicit behavioral and physiological responses comparable to those induced by the stressful event itself. These memories typically readily extinguish when the fear-inducing stimulus is removed [29,30,31] and the primary ways our body naturally mitigates behavioral fear responses are through fear extinction and extinction learning [31,32]. However, the failure of fear extinction, new learning that inhibits fear behavior without erasing the fearful memory [31], is linked to failed coping and psychopathology.

Behavioral freezing is typically used to assess fear learning and memory, with greater time spent freezing being interpreted as stronger fear reactions [33,34,35] and reduced freezing interpreted as extinguished fear and/or attenuated fear memory. Alterations in fear-conditioned freezing after SR, including increases (e.g., [36,37,38,39]) and decreases [40], have been reported previously, depending on the fear paradigm. While freezing is a behavioral manifestation of fear and stress in the CF paradigm, stress-induced hyperthermia (SIH) is an index of the stress response which parallels the time course of stress-induced activation of the hypothalamus–pituitary–adrenal (HPA) axis [41,42]. Together, behavioral freezing and SIH provide a more thorough picture of the stress-induced changes in fear learning and extinction following a sudden, unexpected stressful event. In this study, we assessed behavioral freezing and SIH to better understand the effects of SR, SI and DFS on male and female Wistar rats to determine whether there are sex differences in fear-conditioned behavior and stress responses after exposure to spaceflight hazards. In prior studies, we found that SR and DFS, but not SI, significantly increased freezing in male Wistar strain rats [25,43]. SR and DFS, and SI in some conditions, also significantly increased SIH [44]. However, the effects these stressors have on freezing and SIH between sexes are still unclear.

## 2. Materials and Methods

### 2.1. Subjects

Female and male outbred, retired breeder Wistar rats (8–9 months old upon arrival) from Hilltop Lab Animals, Inc. (Scottdale, PA, USA) served as subjects. The animals arrived in several cohorts. Upon arrival, males and females were housed in separate rooms until the end of the experimental paradigm to account for olfactory-dependent alterations in genetic and hormonal profiles [45,46]. Once the experimental paradigm for one cohort ended, housing rooms were decontaminated and used as a housing rooms for the opposite sex when the next cohort arrived. This was done to account for potential differences between rooms.

Animals were randomly selected for treatment assignment. One subset of animals was individually housed (IH, standard practice for surgically implanted animals in our lab [43,47]) as a control group (SHAM, 14 males and 8–9 females (explained in Section 2.3)) to prevent interference during temperature recordings and potential damage done by cagemates to implanted telemetry transmitters. A separate cohort of animals were IH with opaque barriers between cages (SI, 9 males and 8 females) to prevent visual contact. (It is important to note that SI animals still retained olfactory and auditory perception of other rats in their housing room. Thus, this model may be indicative of visual SI, and the elimination of other sensory perceptions of rodents may result in more exacerbated effects.) SI housing began 35 days post-SR and continued until the end of study. Additional cohorts of rats were sent to the NASA Space Radiation Laboratory (NSRL) in Brookhaven National Laboratory (BNL; Long Island, NY, USA) to receive a one-time dose of SR (15 cGy simplified 5-ion galactic cosmic radiation (GCRsim)). GCRsim consisted of the following ion species: ^1^H (1000 MeV/n, dose rate 1.46–5.80 cGy/min), ^28^Si (600 MeV/n, dose rate 0.09–0.21 cGy/min), ^4^He (250 MeV/n, dose rate 0.15–0.27 cGy/min), ^16^O (350 MeV/n, dose rate 0.17–0.23 cGy/min), ^56^Fe (600 MeV/n, 0.17–0.45 cGy/min), ^1^H (250 MeV/n, dose rate 1.23–4.22 cGy/min). (The BNL staff confirmed spatial beam uniformity and dosimetry.) Irradiated rats were either IH (SR, 10 males and 9 females) or housed in SI conditions (DFS, 11 males and 10 females). Sample sizes were determined based on the availability of resources (e.g., housing cages, animal rooms).

As a control for stress caused by additional handling, transportation, and facility changes, some non-irradiated SHAM (control) and SI animals were shipped to BNL. Ultimately, shipment to BNL produced no significant differences in SHAM or SI freezing behavior compared to animals housed at EVMS [1]. Shipped and in-house groups were combined for statistical analyses.

The animals were housed on a 12:12 light:dark cycle and kept at an ambient temperature of 24.5 °C ± 0.5 °C. Food and water were available ad libitum and Nylabone^®^ chew toys (Nylabone Products, Neptune City, NJ, USA) were provided as enrichment. All experimental procedures were conducted in accordance with the National Institute of Health Guide for the Care and Use of Experimental Animals and were approved by Eastern Virginia Medical School’s Institutional Animal Care and Use Committee (Protocols: 19-018, 22-009).

### 2.2. Surgery

All rats were surgically implanted with wireless telemetry transmitters (ETA F10, Data Sciences International; Minneapolis, MN, USA) at least 3 weeks prior to the start of behavioral testing, as described previously [1,47]. In brief, telemetry monitors were implanted between the scapulae and wires were led to the skull subcutaneously. These transmitters were used to record EEG activity, whole-body activity (not discussed in this manuscript) and temperature. Isoflurane anesthesia (Pivetal Veterinary Supplies; Loveland, CO, USA) was used for all surgeries (inhalant: 5%; induction: 2–3% maintenance). Animals were provided continuous access to ibuprofen (30 mg/kg, oral) for pain relief (24–48 h pre-op, minimum 72 h post-op). Each animal also received subcutaneous injections of penicillin (22,000–100,000 IU/kg, Aspen Veterinary Resources; Liberty, MO, USA), gentamycin (5–8 mg/kg, Aspen Veterinary Resources; Liberty, MO, USA), and dexamethasone (0.5–2 mg/kg, Clipper Distributing Company; St. Joseph, MO, USA) subcutaneously on the day of surgery.

### 2.3. Conditioned Fear Procedures

Fear conditioning was conducted as previously described [25,43]. In brief, conditioned fear training and testing consisted of three phases: shock training (ST), context re-exposure (CTX), and extinction (EXT). All trials occurred during the 4th hour of the light period. SI and DFS animals had their opaque barriers maintained throughout all trials. During ST, animals were placed into a modular shock chamber (23.5 cm × 21 cm × 2.5 cm, 1.27 cm between shock bars; Coulbourn Instruments, Holliston, MA, USA) within an isolation cubicle (52.7 cm × 79.4 cm × 53 cm; Coulbourn Instruments, Holliston, MA, USA) for 30 min, which included a 5 min pre-shock period (to habituate to the chamber), a 20 min shock period (20 footshocks, 0.5 mA, 0.5 s duration, 1 min intertrial intervals), and a 5 min post-shock period. After ST, the animals were returned to their home cages. Seven days after ST, the animals were placed back in the shock chamber for CTX. During CTX, the animals were left in the chamber for 30 min with no footshocks to assess fear learning. After CTX, the animals were returned to their home cages. Fourteen days after CTX (21 days after ST), the animals were placed into the shock chamber for EXT, which lasted 30 min with no footshocks to assess fear extinction. Again, after EXT, the animals were returned to their home cages.

All phases (i.e., ST, CTX, EXT) were video-recorded for visual determination of freezing (lack of body movement excluding respiration [33]). The video recordings were analyzed as previously described [43], in 5 s epochs for the entire 30 min period by a trained individual who was blinded to treatments. Percent freezing was calculated as follows: freezing time/observed time × 100. (Note there were issues with the recording equipment during EXT for one SHAM female. Therefore, their EXT freezing (total and 5 min blocks) was removed from the analyses.)

### 2.4. Temperature Recordings

Telemetry recordings of temperature were conducted as previously described [43]. In brief, recordings took place in the animal’s home cages in their housing rooms. Telemetry receivers were placed underneath the cages, and the implanted transmitters were activated via a magnet. Transmitters were deactivated when the animals were not under study. Twenty-hour recordings were conducted prior to CF (baseline) and immediately following ST, CTX, and EXT. The temperature data (collected in 10 s epochs) from the first 2 h were then compiled to assess SIH.

### 2.5. Statistical Analyses

Investigators were blinded to the treatment groups during statistical analyses. To assess time-dependent alterations in freezing behavior during ST, CTX and EXT, freezing percentages were separated into 5 min blocks and analyzed using three-way analyses of variance (ANOVA) with treatment (SHAM, SI, SR, DFS) and sex (male, female) as the between factors and block as a within factor. The 5 min pre-shock period on the ST day was analyzed separately.

To assess potential freezing changes between CTX and EXT, total 30 min freezing percentages were analyzed by two-way ANOVA with treatment (SHAM, SI, SR, DFS) and sex (male, female) as the between factors.

To assess alterations in SIH, temperature data were separated into 30 min blocks. To assess differences between days, blocks (B1, B2, B3, B4) were analyzed separately using three-way repeated-measures ANOVA with treatment (SHAM, SI, SR, DFS) and sex (male, female) as the between factors and trial (baseline, post-ST, post-CTX, post-EXT) as a within factor. To assess differences over time, trials (baseline, post-ST, post-CTX, post-EXT) were analyzed separately using three-way repeated-measures ANOVA with treatment (SHAM, SI, SR, DFS) and sex (male, female) as between factors and block (B1, B2, B3, B4) as a within factor.

For all analyses, pairwise comparisons were made using Holm–Sidak post hoc tests when indicated by significant ANOVA.

## 3. Results

### 3.1. Freezing During ST

#### 3.1.1. Sex Comparisons

ANOVAs evaluating the differences in freezing percent between sex, treatment and block revealed several main and interaction effects. These findings as well as calculated partial η^2^ values are listed in Supplemental Table S1. Additionally, the averages for each group, standard errors and confidence intervals can be found in Supplemental Table S2. Comparisons within ST revealed that female SHAM froze significantly more than male SHAM during the pre-shock period (Figure 1A). Other treatment groups did not show sex differences in freezing during the pre-shock period (Figure 1B–D). After shock onset, female SHAM froze significantly more than their male counterparts (Figure 1E). Female SR did not show any significant differences compared to male SR (Figure 1G). SI animals had mixed results. Females exposed to isolation independently (SI) froze significantly more than their male counterparts (Figure 1F), but females exposed to SI in combination with SR (DFS) froze significantly less than male DFS (Figure 1H).

#### 3.1.2. Block Comparisons

All sex and treatment groups experienced more freezing during the post-shock blocks (B1–B5) compared to the pre-shock period. Between blocks (after shock onset), males had significantly increased freezing over time in the SHAM and DFS treatment groups, while females had significantly increased freezing over time in the SR and DFS treatment groups (Figure 1E–H). High levels of freezing persisted in all groups after shock presentation had ended (ST5).

#### 3.1.3. Treatment Comparisons

Differences between treatment groups in males and females are shown in Supplemental Figure S1. In brief, females showed no significant differences between treatment groups either pre- or post-shock onset. Males had no significant treatment differences pre-shock; however, post-shock, male SR and DFS froze significantly more than male SHAM and SI (Supplemental Figure S1D), suggesting that SR (alone or DFS) can increase fear responses in males following unexpected stressor exposure.

### 3.2. Freezing During CTX and EXT

#### 3.2.1. Sex Comparisons

The main and interaction effects from the ANOVAs run on freezing percentages during CTX and EXT as well as calculated partial η2 values are listed in Supplemental Table S1 (Averages, standard errors, and confidence intervals can be found in Supplemental Table S2). During CTX, female SHAM and SI froze significantly more than their male counterparts during CTX and EXT (Figure 2A,B,E,F), suggesting that males exposed to isolation have a less severe fear response than females when reintroduced to a previously stressful context. Exposure to radiation produced the opposite effect, with male SR freezing significantly more than female SR during EXT (Figure 2C,G).

#### 3.2.2. Block Comparisons

Across blocks, male SR showed decreased freezing over time during CTX and EXT (Figure 2C,G). Additionally, male DFS showed decreased freezing over time during CTX (Figure 2D). These findings suggest that male animals exposed to SR and DFS show fear extinction throughout the duration of a trial. Apparent decreases in freezing over time in females did not reach significance in any treatment group.

#### 3.2.3. Trial Comparisons

ANOVA comparing total 30 min freezing percentages between CTX and EXT revealed significant main and interaction effects as well as partial η2 values that are listed in Supplemental Table S3. Averages, standard errors and confidence intervals are located in Supplemental Table S4. Between trials, male SHAM and SI showed significant decreases in freezing during EXT compared to CTX (Figure 3A,B), suggesting fear extinction across days. By comparison, male SR and DFS showed no significant change in freezing across days (Figure 3C,D), though both groups showed decreased freezing over time during CTX and/or EXT (see Figure 2). This suggests that SR and DFS experienced reduced fear over time during the recording period (which may be due to acclimation to the shock chamber) but did not experience between-session retention of the extinction memory. There were no significant differences across the CTX and EXT days in any female treatment groups. Differences between treatment groups plotted across 5 min blocks during CTX and EXT for males and females are shown in Supplemental Figure S2. Females showed no differences between treatment groups during CTX; however, female SI froze significantly more than female SR and DFS during block 3 of ETX (Supplemental Figure S2A,C). Male SR and DFS froze significantly more than male SHAM and SI during B1–B4 of CTX and B1–B6 of EXT (Supplemental Figure S2B,D).

### 3.3. Stress-Induced Hyperthermia

Comparisons were made between changes in whole-body temperature and corresponding baseline temperature (Supplemental Table S5). However, neither the between-block (Supplemental Table S6, Supplemental Figure S3) or between-trial (Supplemental Table S7, Supplemental Figure S4) comparisons yielded a significant three-way interaction effect. Therefore, analyses were run on raw temperature values.

#### 3.3.1. Trial Comparisons

Several ANOVAs revealed significant differences between treatment groups, between sexes of the same treatment group and between the different temperature recording trials (baseline, post-ST, post-CTX, post-EXT) in each 30 min recording block (Supplemental Table S8). Averages, standard errors and confidence intervals are included in Supplemental Table S9. All male and female treatment groups experienced SIH, indicated by an increase in whole-body temperature post-ST compared to baseline (Figure 4). Additionally, most animals showed a return to baseline temperatures by the end of the 2 h monitoring period. However, male SR still had significantly elevated temperatures at the end of the post-EXT recording compared to baseline (Figure 4L). Additionally, male DFS had elevated temperatures post-EXT compared to post-CTX (Figure 4N).

#### 3.3.2. Sex Comparisons

There were no significant sex differences in baseline temperature or in SIH responses across days in SHAM rats (Figure 4A–D). Pairwise comparisons revealed that Female SI had significantly higher temperatures at the beginning of the baseline compared to males (Figure 4E). However, by B4 of the baseline recording, female SI had significantly lower temperatures compared to male SI (Figure 4H). Female DFS also had significantly lower temperatures compared to their male counterparts during baseline (Figure 4O). These findings show that females exposed to SI (independently and in combination) may be more prone to alterations in whole-body temperature than males. This trend was not seen post-ST, -CTX or -EXT in SI females. However, animals exposed to radiation had significant differences in whole-body temperature after the CF experiments. Female SR and DFS had significantly lower temperatures compared to their male counterparts post-ST, -CTX and -EXT (Figure 4I–P).

#### 3.3.3. Treatment Comparisons

At the beginning of baseline, male SR and DFS had significantly higher temperatures compared to male SHAM and SI; however, male SR and DFS have lower temperatures compared to male SHAM and SI by the end of baseline (Supplemental Figure S5B). Male SR and DFS return to having higher temperatures than male SHAM and SI post-ST and -EXT (Supplemental Figure S5D,H). Interestingly, female DFS had significantly lower temperatures compared to female SHAM and SR across all recordings, with the most pronounced difference post-CTX (Supplemental Figure S5A,C,E,G).

#### 3.3.4. Baseline Block Comparisons

ANOVAs evaluating comparisons between blocks during baseline, post-ST, post-CTX and post-EXT revealed several main and interaction effects, listed in Supplemental Table S10. Block comparisons during baseline showed no significant differences between male or female SHAM. However, Female SI had significantly higher temperatures during B1 compared to the other three blocks. Male SR had higher temperatures during B1 compared to B3 and B4; however, female SR had higher B1 temperatures compared to the other three blocks. When exposed to DFS, male and females had higher temperatures during B1 compared to their other three blocks (Figure 5A–D).

#### 3.3.5. Post-ST Block Comparisons

Post-ST, male SHAM and SI had higher temperatures during B1 compared to the other three blocks. B2 temperatures were also higher than B3 and B4. Males exposed to radiation (SR and DFS) also had higher temperatures during B3 compared to B4. Female SHAM had higher temperatures during B1 compared to B3 and B4, with B3 temperatures being higher than B4. These differences between blocks were also seen in males and females exposed to SI and DFS; however, females exposed to SR had higher B1 temperatures compared to B4 but not B3. (Figure 5E–H).

#### 3.3.6. Post-CTX Block Comparisons

Both male and female SHAM had significantly higher temperatures during B1 compared to the other three blocks and decreased temperatures during B4 compared to B2 post-CTX. These differences were also found in SI animals. Additionally, temperatures decreased from B1 to B2 in SI males, but not SI females. Males and females exposed to SR had decreased temperatures during B3 and B4 compared to B1 and B2. Female SR had lower temperatures during B2 compared to B1 and during B4 compared to B3. This difference was not seen in male SR. When exposed to DFS, males experience increased temperatures during B1 compared to the other three blocks. However, female DFS experienced steadily decreased temperatures during B1, B2, and B3 (there was no difference between B3 and B4 temperatures) (Figure 5I–L).

#### 3.3.7. Post-EXT Block Comparisons

The temperatures of male SI animals post-EXT were significantly lower during B4 compared to the first two blocks. Males exposed to radiation (SR and DFS) had steadily decreased temperatures until B4 (no significant difference between B3 and B4). Interestingly, differences between blocks were consistent across all female treatment groups. Temperature steadily decreased until B3, with no difference between B3 and B4 (Figure 5M–P).

## 4. Discussion

The current study examined how SI and SR alone and combined (DFS) impacted the effects of fear learning and memories on behavioral freezing and stress responses, as indicated by SIH, in male and female rats. SI, SR and DFS produced changes in freezing that varied between sexes, with greater freezing in females in SHAM and SI groups, whereas SR and DFS increased freezing in males to levels that were similar to those seen in females. SR and DFS also resulted in sex differences in SIH with males showing increased temperatures in most of the ST and fear-recall conditions.

Alterations in fear-conditioned freezing after SR, including increases (e.g., [36,37,38,39]) and decreases [40], and differences between males and females [48] have been reported previously, with differences across fear paradigms. SI in rodents can increase freezing and impair fear extinction compared to normally housed controls [49,50,51]. However, these prior studies used a small number of footshocks and were designed to determine whether SR and SI could impact fear learning and the ability to form fear memories. Thus, these studies provide information on the ability of SR and SI to impair fear learning in brief, transitory stressful situations. By comparison, our work aims to model and build an understanding of how well-established fear memories can lead to persisting changes in behavior that can be resistant to extinction. As such, we have used extensive training procedures with multiple shock presentations, and a distinction should be made between studies examining the ability to form fear memories and our studies, aiming to understand the lasting effects of putatively traumatic stressors. Similar distinctions have been made in the CF literature, where the differential effects of stress [52], extensive training [53] and overtraining [54] on some aspects of fear memory have been reported. Thus, this work should be viewed in the context of the effects of intense and potentially traumatic stress instead of normal learning abilities.

Behavioral freezing has become the standard measure of fear memory in rodent studies [33,55,56], with greater freezing in rodents being considered to indicate greater associative learning [57] as well as stronger fear reactions [33,55,56]. However, ethologically, freezing occurs at intermediate levels of predator threat and it aids in avoiding detection and enhancing perception [58,59,60]. Presenting shock to a confined animal in experimental CF also may make freezing the most likely response [61] by eliminating the potential for defense or escape [62]. Interestingly, the CF paradigm may thus model some aspects of the closed spaceflight environment, as response options to significant unexpected stress or danger may be limited.

Female SHAM and SI rats showed significantly greater freezing in all phases of the CF paradigm than did male SHAM and SI rats. It is also worth noting that, compared to SHAM males, SHAM females showed greater freezing even before the onset of shock and that both SHAM and SI females showed very high levels of freezing after shock onset that continued into the post-shock period. However, compared to SHAM or SI, neither SR nor DFS significantly increased freezing in females. In contrast, both SR and DFS resulted in significant increases in freezing in males during ST, CTX and EXT to levels that approximated those in females and even exceeded levels in females during ST in DFS animals. Thus, interpreting the effects of SR and DFS on freezing requires a consideration of the sex differences that exist prior to irradiation.

Male SR and DFS showed decreased freezing across blocks during CTX and EXT, suggesting that extinction learning may have occurred in these animals. However, there were no significant differences between CTX and EXT in these groups, potentially indicating a lack of significant retention of extinction memory across days or spontaneous recovery, as there were 14 days between CTX and EXT. However, male SHAM and SI showed decreased total freezing behavior from CTX to EXT, indicating that behavioral extinction was retained across exposures. By comparison, females showed no difference between total CTX and EXT freezing or between blocks in any treatment group, suggesting a potential sex difference in the initial extinction learning and later retention of behavioral fear responses.

Female mice have been reported to be resilient to some effects of SR [4,5]. GCRsim-exposed male, but not female, mice exhibited reduced social interactions, increased anxiety-like behavior and impaired recognition memory that were associated with microglia activation, synaptic loss and reductions in the AMPA-expressing synaptic terminals in the hippocampus [5]. It has been hypothesized that molecular changes in the prefrontal cortex and striatum may (in part) contribute to SR-induced changes in memory. Both regions are implicated in conditioned fear. The prefrontal cortex has afferent and efferent projections to the hippocampus (a region implicated in memory formation and consolidation) [63,64,65] and experiences elevated dopamine levels during fear conditioning and extinction [66], while the dorsal and ventral striatum are known to influence fear extinction responses [67,68]. In the context of SR exposure, previous work from Desai et al. [69] has shown that the prefrontal cortex of male mice exposed to GCRsim experienced compromised dopamine neurotransmission. Furthermore, Denisova et al. [70] have shown that SR exposure alters memory signaling through alterations in PRKA levels in the striatum.

SR-induced changes in memory may also be due to heightened neuroimmune responses following SR exposure. Male mice irradiated with low doses of energetic (400 MeV/n) helium ions (^4^He) showed upregulation of Toll-like receptor 4 (TLR4) and increased expression of a pro-inflammatory marker, high-mobility group box 1 protein (HMGB1) [4]. By comparison, non-irradiated female mice had higher basal levels of activated microglia than did non-irradiated male mice, which has been suggested as a possible explanation for the reduced inflammatory response of female mice exposed to cosmic radiation [4]. Sex differences in immune cells begin early in development [71] and continue throughout life with sex-dependent activation of microglia [72], astrocytes [73,74], and cytokine release [75] in response to immune challenge. Differences between male and female responses to stress have been attributed at least partially to differential modulation of inflammatory processes by adrenal and gonadal hormones [21] and these differences have been implicated in the greater vulnerability of females to stress- and fear-based psychiatric disorders (e.g., anxiety and PTSD [21] and depression [21]).

Our current data suggest that there may be a sex-dependent interaction that occurs between SR and relatively intense stress. Based on freezing levels, non-irradiated females appear more responsive to intense stress, but exposure to SR does not appear to further increase freezing levels. By comparison, non-irradiated males show less freezing in response to intense stress, but irradiation increases freezing to levels seen in females. These findings are consistent with females showing greater stress vulnerability but also some resilience to SR and males being more resilient to stress, but more vulnerable to SR. It must be noted that the lack of an increase after freezing in irradiated females could have several interpretations. SHAM and SI females had exceptionally high freezing from the beginning of the shock presentation trials, which thus may have produced a ceiling effect, making it difficult to detect an increase in fear-induced freezing in irradiated females. Alternatively, previous work has shown that females appear more sensitive to experimentally induced pain (like footshock) than males [76]. It may be that SR influenced pain sensitivity in males but not females, which could explain why irradiated males exhibited increased freezing while irradiated females did not. Finally, exposure to footshock in a confined chamber may limit the behavioral response of females. As mentioned previously, the shock chambers, due to their small size, prevent rodents from exhibiting defensive and escape behaviors [61,62], which females have been shown to exhibit as a fear response more than males following stressor exposure [77]. Thus, the lack of change in the fear response in irradiated females may be due to their inability to express other fear behaviors. Future work could assess freezing behavior in an apparatus that allows for the demonstration of other behaviors (e.g., Y-maze) to verify these findings.

We examined SIH as an index of the stress response that parallels the time course of the stress-induced activation of the HPA axis [41,42]. SIH occurs upon stress onset and can persist several hours after the stressor is no longer present [3,78]. All animals experienced a significant increase in body temperature post-ST compared to baseline. Between treatments, males exposed to radiation (SR and DFS) had an initially higher temperature compared to SI during baseline (B1), but over time had decreased temperature compared to SI (B4). Throughout post-ST and -EXT exposures, male SR or DFS had significantly higher temperatures compared to SI at the beginning of the recordings (B1–B3), that eventually returned to baseline, indicating proper SIH resolution over time. Interestingly, females exposed to DFS, but not SR, had significantly lower temperatures than SHAM at the end of the baseline (B4). Additionally, female DFS had significantly lower temperatures compared to SR throughout future recordings. These treatment differences did not resolve over time during CTX (B3–B4). In the context of our freezing data, this further supports the idea that males are more vulnerable to SR. However, DFS exposure appears to have an interactive effect on SIH resolution in females that was not seen in freezing behavior.

Our study shows sex-dependent differences in the effects of spaceflight hazards on fear memory and extinction learning. Between sexes, SHAM male and female rats did not show sex differences in SIH either across days or across time. SI males and females only showed small, but significant, differences during baseline, with females showing increased temperatures in B1 and decreased temperatures in B4. By comparison, SR and DFS males had significantly higher temperatures than their female counterparts during a majority of the 2 h recordings post-ST, -CTX and -EXT. These sex differences suggest that SR and DFS exposure produce a more intense stress response, as indicated by SIH, in males compared to females. Whether this increase in SIH is associated with the increased freezing in irradiated males is not clear. Irradiated and non-irradiated females showed similarly high levels of freezing across days without changes in SIH. We have also found that freezing can extinguish during periods with significantly elevated SIH [44].

While sex comparisons revealed differences in SIH intensity, examination across time within days (blocks) found differences in the time needed for SIH to return to baseline. All male temperatures had noticeably decreased by B2 post-ST; however, differences in female temperatures were not seen until B3. This trend was also seen in SI animals post-CTX but not in any group post-EXT. Taken in combination with the sex differences noted above, it appears that males exposed to SR and DFS experience more intense SIH (indicated by higher maximal temperatures) compared to females following shock and context re-exposures; however, these differences returned to baseline levels quickly. Conversely, females did not experience as high an increase in temperature as males but took longer to return these temperatures to baseline levels. This delayed return to baseline in females was exacerbated by SI.

It is well-documented that spaceflight hazards can impact rodents in a sex-dependent and context-specific manner. Previous research has shown that males exposed to radiation experienced altered short term spatial memory [79,80,81]. However, females did not exhibit this deficit [82]. In comparison, females show deficits in attentional set shifting [83], which could influence their ability to accomplish complex tasks. SR also impacts risk-taking predisposition differently in females and males. Females made more high-risk decisions, while males, although they made low-risk selections, took significantly longer to make decisions [84].

The current findings join the growing evidence from our lab showing that SI and SR, and their combination, may produce neural and behavioral changes that can impact astronaut wellbeing [22,24] and highlight that they can produce sex-dependent and interactive effects. In addition to our findings, work by Kiffer [85] and Wadhwa [86] have shown that different forms of radiation as well as additional spaceflight hazards (e.g., altered gravity) can also produce interactive effects. Furthering our understanding of the ways that spaceflight stressors interact with one another is critical for future mitigation methods, as current intervention strategies primarily address one stressor independently but not how these stressors interact with one another [87,88]. Due to the complex interplay between spaceflight hazards, it may be useful to integrate advanced modelling approaches, as they would allow us to better understand the wholistic impacts of their combined effects on the human body. By understanding how these stressors impact systems differently when applied together, we may be able to develop more advanced mitigation strategies that can better protect astronauts as they journey further into space.

During spaceflight, SI can induce physical deficits that may impact performance (e.g., decreased sensorimotor function, impaired immune response) [1,24,89,90] and psychological deficits that may impact team dynamics (e.g., emotional bluntness, depression) [91,92,93]. SR exposure can impact several components of mission performance (e.g., thermoregulation, sensorimotor function, memory, executive function, stress neurocircuitry) [47,94,95,96,97,98]. Beyond the immediate effects of SI and SR, both spaceflight stressors can have long-lasting effects on the body. Individuals exposed to SI had a higher likelihood of developing high blood pressure, cardiovascular disease and type II diabetes [99,100,101,102]. Both human and rodent studies have shown that SR exposure increases the risk of later developing cancer, Alzheimer’s Disease, Parkinson’s Disease and dementia [24,98,103,104,105,106,107,108,109]. Thus, it is critical to determine the effects of SR and SI, as well as other spaceflight hazards, and their interactions in order to understand factors that impact mission health and performance as well as their long-term effects.

In summary, these data demonstrate that male and female rats can exhibit different baseline fear learning and stress responses to significant stressful challenges which can be differentially impacted by spaceflight hazards. Baseline differences suggest that males and females may have sex-specific characteristics attuned to their behavioral capacities or needs, whereas differences after exposure to spaceflight hazards suggest sex differences in stress vulnerability. In general, data suggest that understanding both between- and within-sex effects will be important for assessing how spaceflight hazards impact performance and developing mitigation strategies that may need to be tailored for males and females. In the context of our findings on stress responses and extinction learning, screening for stress vulnerability prior to mission onset (using behavioral (e.g., Trier Social Stress Test, Maastricht Acute Stress Test) and physiological tests (e.g., wearable biomonitors to assess skin conductance and heart rate variability, urine samples to assess cortisol levels)) could help develop personalized countermeasures for male and female astronauts.

## Figures and Tables

**Figure 1:**
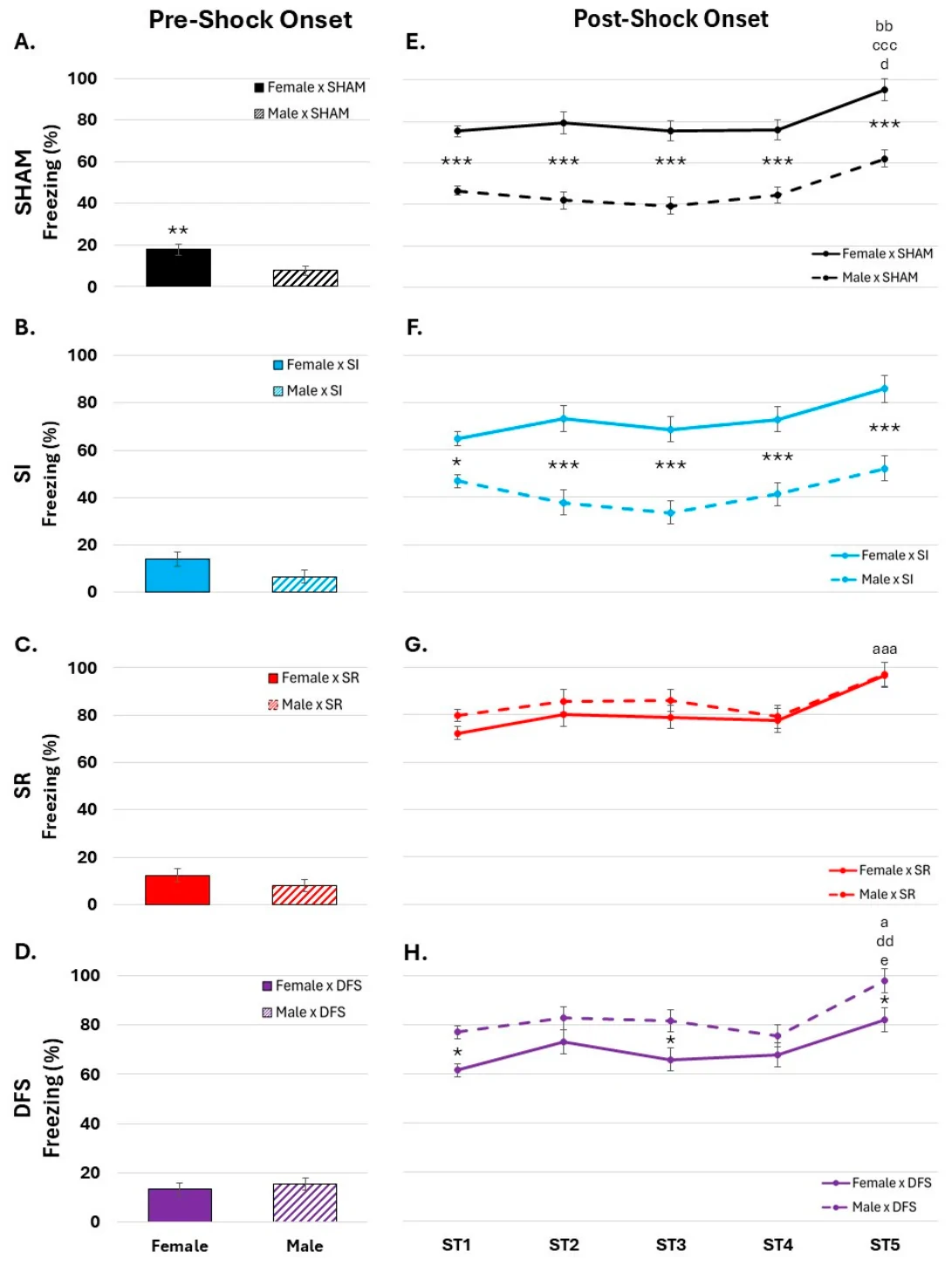
Differences in freezing behavior (**A**–**D**) before and (**E**–**H**) after shock onset in female and male SHAM, social isolation (SI), space radiation (SR) and dual flight stressor (DFS) rats. *, *p* < 0.05, **, *p* < 0.01, ***, *p* < 0.001, females compared to male counterparts; a, *p* < 0.05; aaa, *p* < 0.001, compared to B1 in males; bb, *p* < 0.01, compared to B2 in males; ccc, *p* < 0.001, compared to B3 in males; d, *p* < 0.05; dd, *p* < 0.01, compared to B4 in males; e, *p* < 0.05, compared to B1 in females.

**Figure 2:**
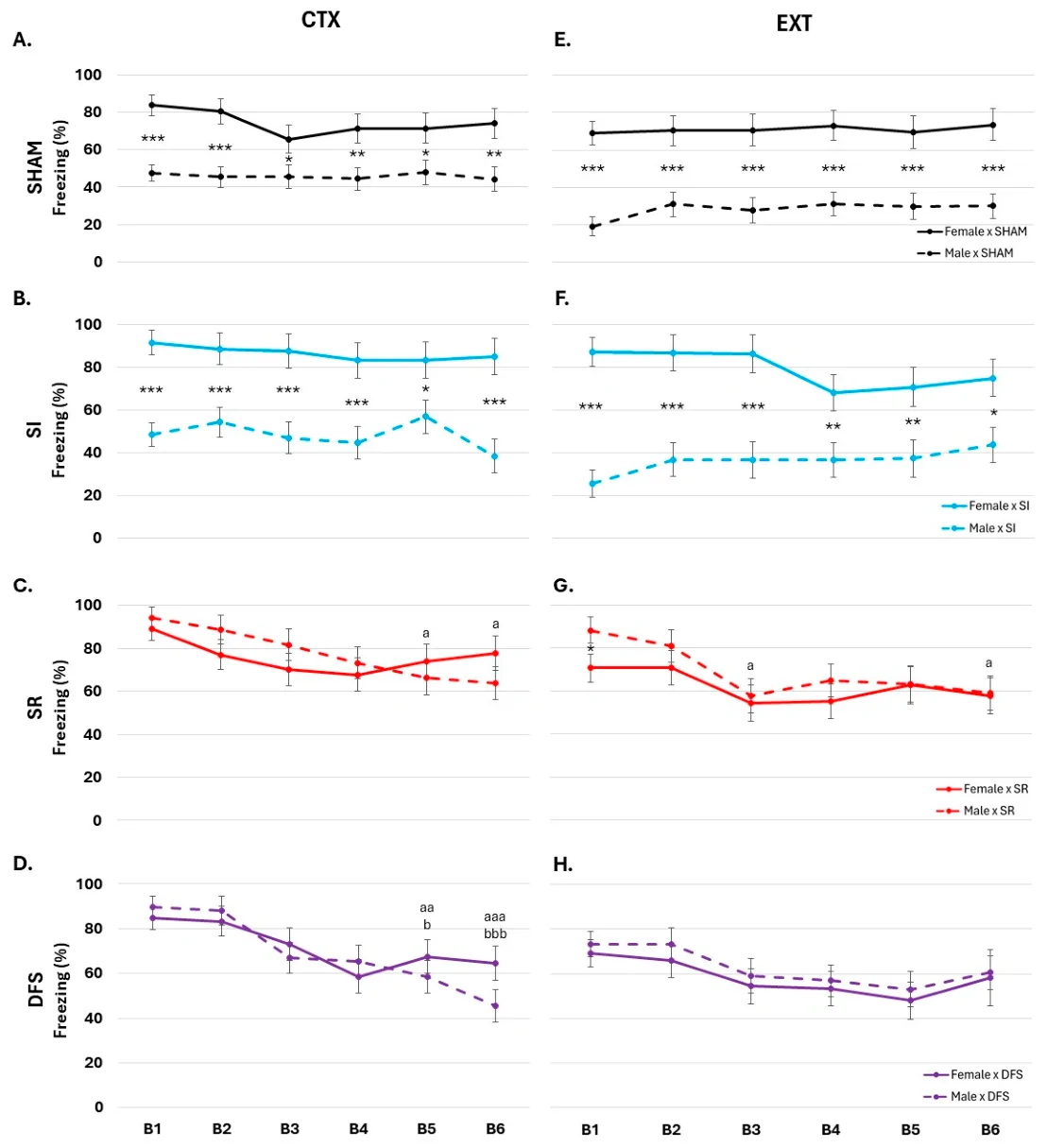
Differences in freezing behavior during (**A**–**D**) CTX and (**E**–**H**) EXT in female and male SHAM, social isolation (SI), space radiation (SR) and dual flight stressor (DFS) rats. *, *p* < 0.05; **, *p* < 0.01; ***, *p* < 0.001, females compared to male counterparts; a, *p* < 0.05; aa, *p* < 0.01; aaa, *p* < 0.001, compared to B1 in males; b, *p* < 0.05; bbb, *p* < 0.001, compared to B2 in males.

**Figure 3:**
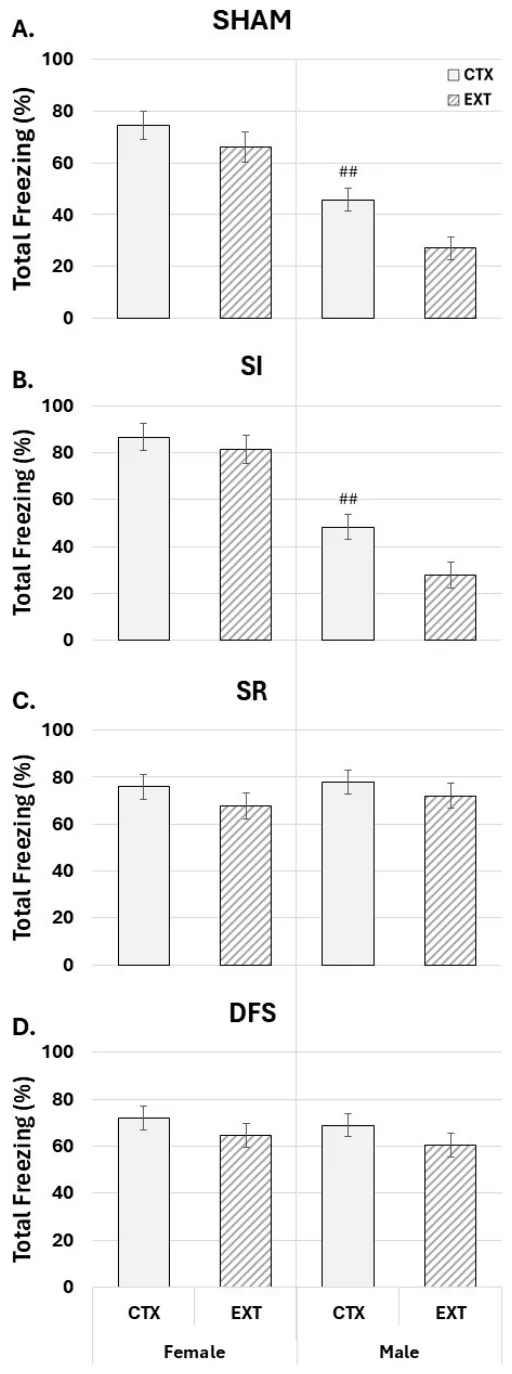
Differences in freezing behavior between CTX and EXT in female and male (**A**) SHAM, (**B**) social isolation (SI), (**C**) space radiation (SR) and (**D**) dual flight stressor (DFS) rats. ##, *p* < 0.01, compared to EXT.

**Figure 4:**
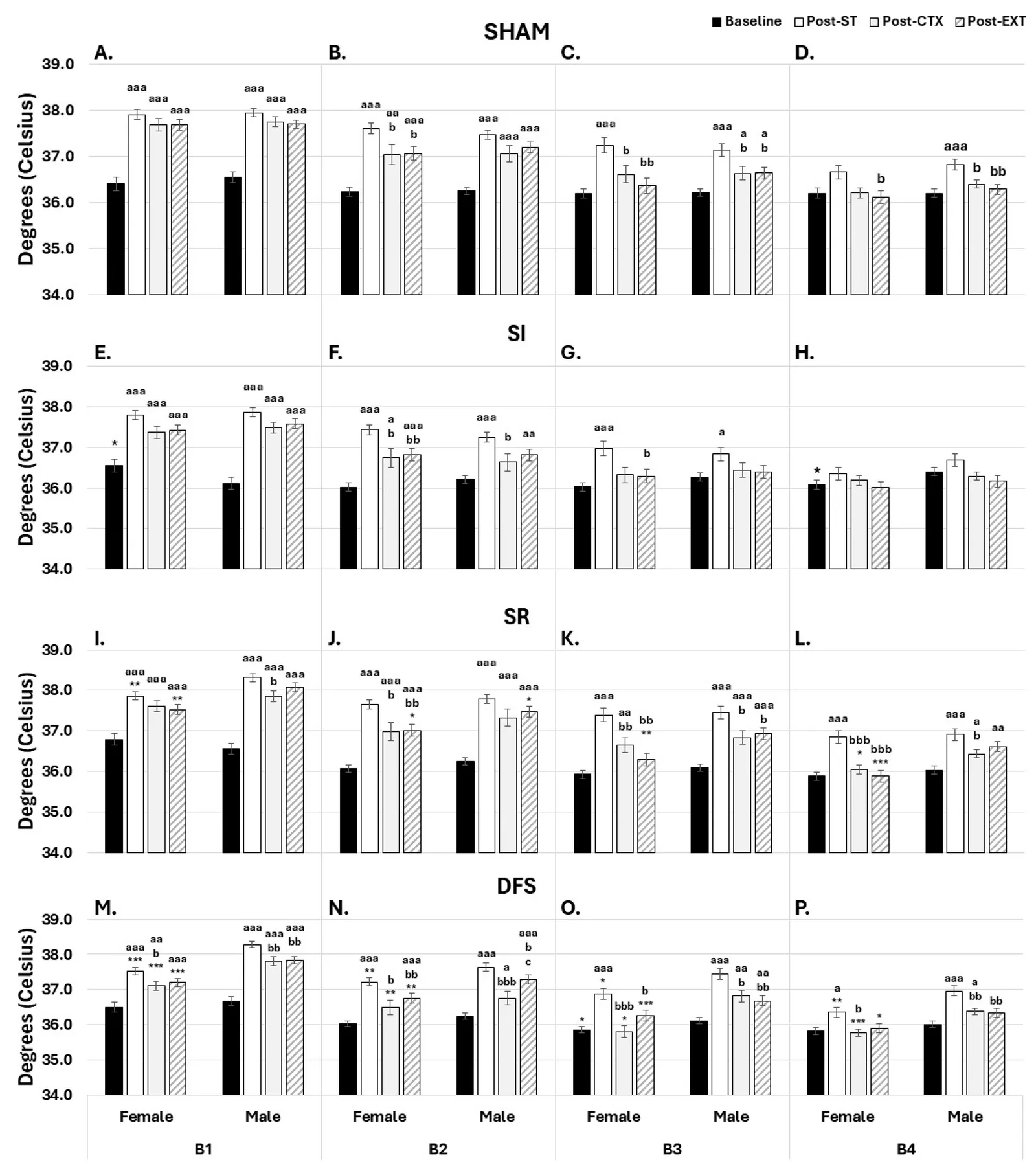
Differences in temperature in 30 min blocks during baseline, post-ST, post-CTX and post-EXT in female and male (**A**–**D**) SHAM; (**E**–**H**) social isolation (SI); (**I**–**L**) space radiation (SR); and (**M**–**P**) dual flight stressor (DFS) rats. *, *p* < 0.05; **, *p* < 0.01; ***, *p* < 0.001, females compared to male counterparts; a, *p* < 0.05; aa, *p* < 0.01; aaa, *p* < 0.001, compared to baselines of the same sex; b, *p* < 0.05; bb, *p* < 0.01; bbb, *p* < 0.001, compared to post-ST of the same sex; c, *p* < 0.05, compared to post-CTX of the same sex.

**Figure 5:**
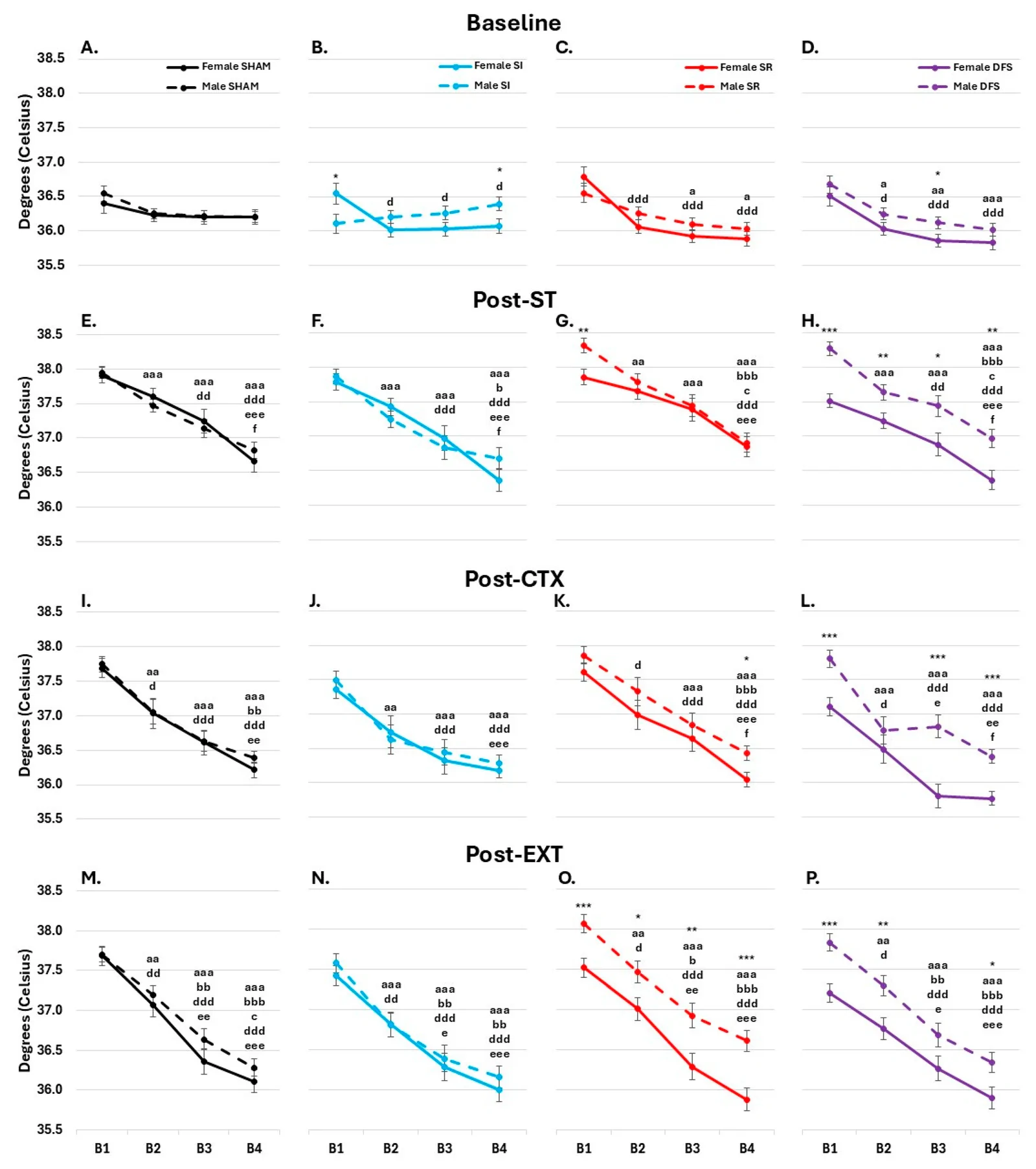
Differences in temperature across blocks during (**A**–**D**) baseline, (**E**–**H**) post-ST, (**I**–**L**) post-CTX and (**M**–**P**) post-EXT in SHAM, social isolation (SI), space radiation (SR) and dual flight stressor (DFS) rats. *, *p* < 0.05; **, *p* < 0.01; ***, *p* < 0.001, females compared to male counterparts; a, *p* < 0.05; aa, *p* < 0.01; aaa, *p* < 0.001, compared to B1 in males; b, *p* < 0.05; bb, *p* < 0.01; bbb, *p* < 0.001, compared to B2 in males; c, *p* < 0.05, compared to B3 in males; d, *p* < 0.05; dd, *p* < 0.01; ddd, *p* < 0.001, compared to B1 in females; e, *p* < 0.05; ee, *p* < 0.01; eee, *p* < 0.001, compared to B2 in females; f, *p* < 0.05, compared to B3 in females.

## Data Availability

Experimental data are available upon request.

## Supplementary Materials

The following supporting information can be downloaded at: https://www.mdpi.com/article/10.3390/life16101638/s1, Figure S1: Differences in freezing behavior before and after shock onset between male and female SHAM, social isolation (SI), space radiation (SR) and dual flight stressor (DFS) rats; Figure S2: Differences in freezing behavior during CTX and EXT between male and female SHAM, social isolation (SI), space radiation (SR) and dual flight stressor (DFS) rats; Figure S3: Sex differences in whole-body temperatures post-ST, post-CTX and post-EXT compared to baseline in female and male SHAM, social isolation (SI), space radiation (SR) and dual flight stressor (DFS) rats; Figure S4: Treatment differences in whole-body temperatures post-ST, post-CTX and post-EXT compared to baseline in female and male SHAM, social isolation (SI), space radiation (SR) and dual flight stressor (DFS) rats; Figure S5: Differences in whole-body temperature in 30 min blocks during baseline, post-ST, post-CTX and post-EXT in female and male SHAM, social isolation (SI), space radiation (SR) and dual flight stressor (DFS) rats; Table S1: Three-way ANOVA tables showing results from analyzing differences in freezing behaviors between 5 min blocks in female and male SHAM, social isolation (SI), space radiation (SR) and dual flight stressor (DFS) rats during ST, CTX and EXT; Table S2: Average freezing percentages, standard errors and confidence intervals (by block) from female and male SHAM, social isolation (SI), space radiation (SR) and dual flight stressor (DFS) rats during ST, CTX and EXT; Table S3: Results from the three-way ANOVA analyzing differences between total 30 min freezing percentages during CTX and EXT in female and male SHAM, social isolation (SI), space radiation (SR) and dual flight stressor (DFS) rats; Table S4: Average total freezing percentages, standard errors and confidence intervals from female and male SHAM, social isolation (SI), space radiation (SR) and dual flight stressor (DFS) rats during CTX and EXT; Table S5: Average change in whole-body temperature compared to baseline, standard error and confidence intervals (by block) in females and male SHAM, social isolation (SI), space radiation (SR) and dual flight stressor (DFS) rats post-ST, post-CTX and post-EXT; Table S6: Three-way ANOVA tables showing results from analyzing differences in whole-body temperature compared to baseline between 30 min blocks in female and male SHAM, social isolation (SI), space radiation (SR) and dual flight stressor (DFS) rats post-ST, post-CTX and post-EXT; Table S7: Three-way ANOVA tables showing results from analyzing differences in whole-body temperature compared to baseline between trials (post-ST, post-CTX, post-EXT) in female and male SHAM, social isolation (SI), space radiation (SR) and dual flight stressor (DFS) rats during B1, B2, B3 and B4; Table S8: Three-way ANOVA tables showing results from analyzing differences in raw whole-body temperature between trials (baseline, post-ST, post-CTX, post-EXT) in female and male SHAM, social isolation (SI), space radiation (SR) and dual flight stressor (DFS) rats post-ST, post-CTX and post-EXT; Table S9: Average raw whole-body temperature, standard errors and confidence intervals (by block) from female and male SHAM, social isolation (SI), space radiation (SR) and dual flight stressor (DFS) rats during baseline, post-ST, post-CTX and post-EXT; Table S10: Three-way ANOVA tables showing results from analyzing differences in raw whole-body temperature between trials (baseline, post-ST, post-CTX, post-EXT) in female and male SHAM, social isolation (SI), space radiation (SR) and dual flight stressor (DFS) rats during B1, B2, B3 and B4.
